# (Un)Healthy in the City: Respiratory, Cardiometabolic and Mental Health Associated with Urbanity

**DOI:** 10.1371/journal.pone.0143910

**Published:** 2015-12-02

**Authors:** Wilma L. Zijlema, Bart Klijs, Ronald P. Stolk, Judith G. M. Rosmalen

**Affiliations:** 1 University of Groningen, University Medical Center Groningen, Department of Epidemiology, Groningen, The Netherlands; 2 University of Groningen, University Medical Center Groningen, Department of Psychiatry, Groningen, The Netherlands; Hasselt University, BELGIUM

## Abstract

**Background:**

Research has shown that health differences exist between urban and rural areas. Most studies conducted, however, have focused on single health outcomes and have not assessed to what extent the association of urbanity with health is explained by population composition or socioeconomic status of the area. Our aim is to investigate associations of urbanity with four different health outcomes (i.e. lung function, metabolic syndrome, depression and anxiety) and to assess whether these associations are independent of residents’ characteristics and area socioeconomic status.

**Methods:**

Our study population consisted of 74,733 individuals (42% males, mean age 43.8) who were part of the baseline sample of the LifeLines Cohort Study. Health outcomes were objectively measured with spirometry, a physical examination, laboratory blood analyses, and a psychiatric interview. Using multilevel linear and logistic regression models, associations of urbanity with lung function, and prevalence of metabolic syndrome, major depressive disorder and generalized anxiety disorder were assessed. All models were sequentially adjusted for age, sex, highest education, household equivalent income, smoking, physical activity, and mean neighborhood income.

**Results:**

As compared with individuals living in rural areas, those in semi-urban or urban areas had a poorer lung function (β -1.62, 95% CI -2.07;-1.16), and higher prevalence of major depressive disorder (OR 1.65, 95% CI 1.35;2.00), and generalized anxiety disorder (OR 1.58, 95% CI 1.35;1.84). Prevalence of metabolic syndrome, however, was lower in urban areas (OR 0.51, 95% CI 0.44;0.59). These associations were only partly explained by differences in residents’ demographic, socioeconomic and lifestyle characteristics and socioeconomic status of the areas.

**Conclusions:**

Our results suggest a differential health impact of urbanity according to type of disease. Living in an urban environment appears to be beneficial for cardiometabolic health but to have a detrimental impact on respiratory function and mental health. Future research should investigate which underlying mechanisms explain the differential health impact of urbanity.

## Introduction

There’s no place like home. But is this place or home a healthy one? In the present-day, more than half of the world’s population lives in urban areas [1], but this may not be the healthiest environment to live in. Living in urban areas exposes people to traffic fumes, noise, crime, and other stressful factors associated with city living. On the other hand, urban living may also be beneficial because of a variety of food choices, proximity of health care services, and other facilities such as sports amenities.

Indeed, research suggests that there are urban-rural health differences. Living in a less urbanized or rural environment rather than an urban environment is for example associated with increased blood pressure [2,3], obesity [4,5], and higher cholesterol levels [6]. However, living in more densely populated areas may deteriorate respiratory [7] and mental health [8], and may be a risk factor for breast cancer [9,10]. Urban-rural differences in mortality have also been observed. For example, positive relations between population density and mortality from lung cancer, chronic obstructive pulmonary disease and ischemic heart disease have been observed [11], while a beneficial effect of high urbanity on all-cause mortality has also been reported [12].

Although urban-rural health differences have been a popular research topic in the past 10 years, much is still to gain in the area of research. A recent review identified areas in which more research is needed [13]. For example, some of the previous studies used self-report outcome measures [13]. It has been shown that self-report of for example height and weight can lead to underestimation of overweight [14], indicating the importance of objectively measured outcomes. Furthermore, in addition to individual socioeconomic status, neighborhood level socioeconomic status should also be taken into account. Neighborhood socioeconomic status is related to the health of its residents and also to neighborhood characteristics including availability of services, infrastructure and social cohesion, which in turn influence health [15]. Previous studies often investigated one single health outcome. Because city living can have both negative and positive effects on health, we think it is important to study multiple health outcomes in relation to urbanity. We studied lung function, metabolic syndrome, depression and anxiety. The burden of disease is high for each of these diseases [16], and previous research has shown that they may be related to the degree of urbanity. Our aim is to investigate associations of urbanity with four different health outcomes (i.e. lung function, metabolic syndrome, depression and anxiety) and to assess whether these associations are independent of individual characteristics and area socioeconomic status.

## Methods

### Study design

LifeLines is a multi-disciplinary prospective population-based cohort study examining in a unique three-generation design the health and health-related behaviors of 167,729 persons living in the North East region of The Netherlands. It employs a broad range of investigative procedures in assessing the biomedical, socio-demographic, behavioral, physical and psychological factors which contribute to the health and disease of the general population, with a special focus on multimorbidity and complex genetics [17]. Recruitment of study participants took place between 2006–2013 through general practitioners and self-enrollment. Participants visited the research sites for a physical examination, including spirometry, anthropometry, collection of fasting blood samples, and a psychiatric interview. An extensive questionnaire was completed at home [18].

### Participants

At the time of analysis, recruitment of participants was still ongoing. The present study is therefore a cross-sectional analysis based on the first data release of 95,432 participants, of which 74,733 had complete data on degree of urbanity. Participants were excluded in the corresponding analyses when they had missing data on the variables needed to define the outcome, resulting in complete data for 63,946 (lung function), 73,278 (metabolic syndrome), or 71,536 (MDD and GAD) participants. All participants provided written informed consent. The study protocol was carried out in accordance to the Declaration of Helsinki, and approved by the medical ethical review committee of the University Medical Center Groningen.

### Degree of urbanity and neighborhood socioeconomic status

Degree of urbanity and neighborhood level socioeconomic status were obtained from Statistics Netherlands [19]. Home addresses of study participants were geocoded and subsequently the corresponding neighborhood was determined. This resulted in 2,524 unique neighborhoods for the current dataset. Degree of urbanity was expressed as the address density (average number of addresses per square kilometer within a range of 1 kilometer), and was categorized into five levels ranging from rural (<500 addresses per km^2^; e.g. very small villages) to urban (≥2500 addresses per km^2^; e.g. city center of a provincial capital) [19]. Neighborhood socioeconomic status was operationalized as neighborhood income (net income per year divided by number of inhabitants in the neighborhood in euros). Because of privacy reasons, data on neighborhood income were only made available for neighborhoods with a minimum of 200 inhabitants [19]. For the current dataset, this resulted in 1,176 missing observations.

### Metabolic syndrome

Participants were considered to have metabolic syndrome if they satisfied at least three out of the five criteria according to the revised National Cholesterol Education Program’s Adult Treatment Panel III (NCEP-ATPIII) [20]. The criteria are as follows: (1) systolic blood pressure (SBP) ≥130 mmHg or diastolic blood pressure (DBP) ≥85 mmHg or use of blood pressure-lowering medication; (2) fasting blood glucose level ≥5.6 mmol/l or use of blood glucose-lowering medication or diagnosis of type 2 diabetes; (3) high-density lipoprotein(HDL)-cholesterol <1.03 mmol/l in men or <1.30 mmol/l in women or lipid-lowering medical treatment; (4) triglycerides ≥1.70 mmol/l or medication for elevated triglycerides; (5) and waist circumference ≥102 cm in men or ≥88 cm in women. If body mass index (BMI) is ≥30 kg/m^2^, abdominal obesity was assumed and waist circumference is not included as a criterion. When BMI is ≥30 kg/m^2^, at least two out of four other criteria should be met to fulfill the criteria for metabolic syndrome.

Anthropometric and blood pressure measurements were conducted by trained medical professionals using a standardized protocol. Fasting blood samples were collected and analyzed on the day of collection, and medication use was assessed by means of a questionnaire.

### Lung function

FEV1 (forced expiratory volume in 1 second) was measured with spirometry during a medical examination performed in a standardized setting following American Thoracic Society guidelines. Lung function varies with age, height, sex and ethnicity. Therefore, lung function was compared to predicted values, which were calculated with equations from the European Respiratory Society [21]. Subsequently, the percentage FEV1 relative to the predicted values (FEV1% predicted) was calculated. Since the number of complete cases for the lung function analyses were lower compared to the analyses for the other health outcomes, we imputed the missing values using a Markov Chain Monte Carlo algorithm in IBM SPSS Statistics (version 22). All variables that are used in the lung function analyses were used as predictors in the imputation model to improve imputation results. Analyses were undertaken with the original data and the imputed dataset. Analyses on the imputed dataset did not result in different conclusions compared to analyses on the original dataset. This indicates that the missing values probably had no effect on our results, and therefore results based on the original dataset are reported.

### Major depressive disorder and generalized anxiety disorder

For the assessment of major depressive disorder (MDD) and generalized anxiety disorder (GAD), participants were interviewed by trained medical professionals during their visit to the research facilities. MDD and GAD were assessed with the Mini-International Neuropsychiatric Interview (MINI). The MINI is a brief structured interview for diagnosing psychiatric disorders as defined by the DSM-IV (Diagnostic and Statistical Manual of Mental Disorders) and ICD-10 (International Statistical Classification of Diseases and Related Health Problems).

### Covariates

Sex, age, highest obtained educational degree, household income, number of persons in the household, current smoking and physical activity were assessed with a questionnaire. Household equivalent income was calculated as net household income per month divided by the square root of the number of persons in the household [22]. Participants were current smokers if they indicated current smoking or smoking in the last month. Physical activity was assessed by asking how many days per week participants are physically active for at least half an hour.

### Statistical analysis

Sample characteristics were described with means (with standard deviations) and prevalence (%) stratified for degree of urbanity. Because of non-normal distribution of neighborhood income medians and interquartile ranges (IQRs) were calculated instead. To account for the within-neighborhood correlation of the outcomes, associations were analyzed with multilevel analysis with a random intercept defined at the neighborhood level. First, associations of urbanity (reference category rural) and lung function, metabolic syndrome, MDD and GAD were assessed. Models were subsequently adjusted for neighborhood income (model 2); age, sex (model 3), education, household equivalent income (model 4); smoking, and physical activity (model 5). Adjusting for these covariates enabled us to assess whether neighborhood income, individual characteristics, socioeconomic characteristics, and lifestyle factors could explain (part of) the association of urbanity and health. Outcomes were expressed as regression coefficients (β) for FEV1% predicted, and as odds ratios (ORs) for metabolic syndrome, MDD and GAD. Model comparison was based on the change in log-likelihood. Multilevel models were analyzed with R (version 3.0.1) using the lme4 package (version 1.1–7) [23]. Associations were considered statistically significant if the 95% confidence intervals (CI) did not include zero (β) or one (OR).

## Results

### Population characteristics

Of the 74,733 participants in the study sample, 42% were male, the majority lived in rural neighborhoods (41%) and the mean age was 43.8 (SD 11.6) years. Mean FEV1% predicted was 102.4; 15.4% were classified as having metabolic syndrome, MDD and GAD prevalence was respectively 2.3% and 4.5% (Table 1). The lowest average FEV1% predicted and highest prevalence of MDD was observed in semi-urban neighborhoods. Highest prevalence of metabolic syndrome was observed in intermediate urban-rural neighborhoods, and highest GAD prevalence in urban neighborhoods. Participants living in urban neighborhoods were on average younger, more highly educated, more often current smokers, and slightly more often physically active.

**Table 1:** Population characteristics of the total study population (n = 74,733) and stratified by urbanity.

		Total	Rural	Semi-rural	Intermediate urban-rural	Semi-urban	Urban
N		74,733	30,823	18,515	12,813	7,745	4,837
Males (%)		42.0	42.6	43.1	40.8	40.8	38.9
Age, mean (SD)		43.8 (11.6)	44.8 (11.1)	44.2 (10.7)	44.2(11.5)	42.0 (12.1)	36.1 (11.8)
Education level (%)	No or primary	2.1	2.1	2.0	2.2	3.1	1.3
	Lower or preparatory vocational	13.1	15.1	12.7	12.4	13.2	3.7
	Lower general secondary	13.5	14.7	13.9	13.8	12.9	5.6
	Intermediate vocational or apprenticeship	31.7	34.4	32.2	32.0	29.4	15.8
	Higher general secondary or pre-university secondary	9.0	8.0	9.0	9.3	9.1	15.2
	Higher vocational or university	30.4	25.8	30.2	30.3	32.3	58.3
Household equivalent income/month (%)	<€1000	18.0	17.4	15.7	18.1	21.7	23.9
	€1000–€1300	22.4	23.7	21.1	22.6	23.2	17.5
	€1300–€1600	16.7	18.4	17.9	17.0	13.8	7.3
	€1600–€1900	21.4	20.2	24.8	20.7	19.7	20.5
	>€1900	21.4	20.2	20.4	21.7	21.6	30.8
Current smokers (%)		22.8	21.5	21.5	23.4	27.6	27.2
Days PA, mean (SD)		4.3 (2.2)	4.2 (2.2)	4.2 (2.2)	4.3 (2.2)	4.3 (2.2)	4.5 (2.2)
FEV1% predicted, mean (SD)		102.4 (13.0)	103.0 (13.1)	102.6 (13.0)	101.9(13.2)	101.5 (12.9)	102.1(12.4)
Metabolic syndrome (%)		15.4	15.9	16.1	16.5	15.0	8.1
GAD (%)		4.5	3.8	4.2	5.6	5.5	5.8
MDD (%)		2.3	2.0	2.3	2.9	3.2	2.4
Neighborhood income (x1000 euro), median (IQR)		19.0 (3.3)	19.1 (2.6)	19.4 (3.3)	19.1 (3.2)	18.3 (3.6)	18.6 (4.8)

Abbreviations: SD: standard deviation; IQR: interquartile range; FEV1% predicted: forced expiratory volume in 1 second relative to the predicted value; MDD: major depressive disorder; GAD: generalized anxiety disorder; days PA: number of days physically active

### Multilevel models with random intercept for neighborhood

#### Lung function

Living in urban, semi-urban and intermediate urban-rural neighborhoods was associated with a decrease in lung function, when compared to living in rural neighborhoods. The largest association was observed for semi-urban neighborhoods, with a decrease in lung function of -1.62% (95% CI -2.07;-1.16), followed by intermediate urban-rural neighborhoods (β -1.06, 95% CI -1.45;-0.67), and urban (β -0.99, 95% CI -1.52;-0.47) (Table 2). The gradient of regression coefficients indicates a quadratic (concave) relationship between urbanity and lung function. The association for semi-rural (vs. rural) was not statistically significant. Adjustment for neighborhood income resulted in both slight increases and decreases in the regression coefficients for the urbanity categories; this was also true for additional adjustment for demographic and SES variables, as well as for smoking and physical activity. In the fully adjusted model, negative associations of urbanity and lung function were still observed, except for the urban category, which was no longer statistically significant. However, model comparison based on log likelihood ratio tests indicated that model 5 did not improve significantly over model 4. Participants in neighborhoods of higher urbanity were more often current smokers, and this may influence the association between urbanity and lung function.

**Table 2:** Associations of urbanity and lung function (FEV1% predicted) estimated from linear multilevel models (n = 63,946).

	Model 1		Model 2		Model 3		Model 4		Model 5	
	β	95% CI	β	95% CI	β	95% CI	β	95% CI	β	95% CI
Urbanity										
Rural	reference		reference		reference		reference		reference	
Semi-rural	-0.32	-0.67;0.02	-0.48	-0.81;-0.15	-0.39	-0.71;-0.06	-0.51	-0.82;0.18	-0.59	-0.91;-0.26
Intermediate urban-rural	-1.06	-1.45;-0.67	-1.08	-1.44;-0.72	-1.01	-1.37;-0.65	-1.13	-1.48;-0.77	-1.11	-1.47;-0.75
Semi urban	-1.62	-2.07;-1.16	-1.56	-1.99;-1.13	-1.26	-1.69;-0.83	-1.37	-1.80;-0.95	-1.17	-1.61;-0.74
Urban	-0.99	-1.52;-0.47	-0.88	-1.38;-0.39	-0.01	-0.51;0.49	-0.66	-1.16;-0.15	-0.48	-1.01;0.04

Abbreviations: FEV1% predicted: forced expiratory volume in 1 second relative to the predicted value; 95% CI: 95% confidence interval, model 1: unadjusted. model 2: adjusted for neighborhood income. model 3: adjusted for neighborhood income, sex, age. model 4: adjusted for neighborhood income, sex, age, education, household equivalent income. model 5: adjusted for neighborhood income, sex, age, education, household equivalent income, smoking, physical activity.

#### Metabolic syndrome

Living in an urban neighborhood was associated with lower odds for metabolic syndrome (OR 0.51, 95% CI 0.44;0.59), when compared to living in rural neighborhoods (Table 3). Associations for other urbanity categories (vs. rural) were not statistically significant. Associations remained similar after adjustment for neighborhood income. When additionally adjusted for demographic and SES variables, the OR for urban (vs. rural) for metabolic syndrome became 0.81 (95% CI 0.71;0.93), indicating attenuation of the association. After adjusting for smoking and physical activity, associations of urbanity and metabolic syndrome remained similar. Participants in neighborhoods of higher urbanity were more often physically active, but this did not explain the association between urbanity and metabolic syndrome.

**Table 3:** Associations of urbanity and metabolic syndrome estimated from logistic multilevel models (n = 73,278).

	Model 1		Model 2		Model 3		Model 4		Model 5	
	OR	95% CI	OR	95% CI	OR	95% CI	OR	95% CI	OR	95% CI
Urbanity										
Rural	reference		reference		reference		reference		reference	
Semi-rural	0.96	0.88;1.04	0.98	0.90;1.06	1.02	0.94;1.10	1.05	0.97;1.13	1.05	0.97;1.13
Intermediate urban-rural	1.04	0.95;1.15	1.03	0.95;1.13	1.08	0.98;1.18	1.10	1.01;1.20	1.09	1.00;1.19
Semi urban	0.94	0.84;1.05	0.92	0.83;1.03	1.03	0.93;1.15	1.06	0.95;1.17	1.04	0.94;1.16
Urban	0.51	0.44;0.59	0.49	0.43;0.56	0.69	0.60;0.79	0.81	0.71;0.93	0.80	0.70;0.92

Abbreviations: OR: odds ratio; 95% CI: 95% confidence interval. model 1: unadjusted. model 2: adjusted for neighborhood income. model 3: adjusted for neighborhood income, sex, age. model 4: adjusted for neighborhood income, sex, age, education, household equivalent income model 5: adjusted for neighborhood income, sex, age, education, household equivalent income, smoking, physical activity.

#### Major depressive disorder

Living in intermediate urban-rural (OR 1.39, 95% CI 1.17;1.65) and semi-urban neighborhoods (OR 1.65, 95% CI 1.35;2.00) was associated with higher odds for MDD, compared to living in rural neighborhoods (Table 4). Associations for other urbanity categories (vs. rural) were not statistically significant. Also here, associations remained similar after adjustment for neighborhood income. However, additional adjustment for demographic and SES variables resulted in amplification of the associations of degree of urbanity and MDD. ORs increased and became statistically significant for all urbanity categories. When additionally adjusted for smoking and physical activity, results remained similar.

**Table 4:** Associations of urbanity and major depressive disorder estimated from logistic multilevel models (n = 71,536).

	Model 1		Model 2		Model 3		Model 4		Model 5	
	OR	95% CI	OR	95% CI	OR	95% CI	OR	95% CI	OR	95% CI
Urbanity										
Rural	reference		reference		reference		reference		reference	
Semi-rural	1.08	0.92;1.27	1.16	1.00;1.36	1.16	1.00;1.62	1.22	1.05;1.42	1.21	1.04;1.40
Intermediate urban-rural	1.39	1.17;1.65	1.58	1.19;1.64	1.38	1.18;1.62	1.44	1.23;1.69	1.43	1.22;1.67
Semi urban	1.65	1.35;2.00	1.40	1.31;1.90	1.56	1.29;1.87	1.60	1.33;1.93	1.54	1.29;1.85
Urban	1.26	0.99;1.59	1.16	0.93;1.46	1.12	0.89;1.40	1.47	1.17;1.86	1.42	1.12;1.78

Abbreviations: OR: odds ratio; 95% CI: 95% confidence interval. model 1: unadjusted. model 2: adjusted for neighborhood income. model 3: adjusted for neighborhood income, sex, age. model 4: adjusted for neighborhood income, sex, age, education, household equivalent income. model 5: adjusted for neighborhood income, sex, age, education, household equivalent income, smoking, physical activity.

#### Generalized anxiety disorder

We observed that living in urban (OR 1.58, 95% CI 1.35;1.84), semi-urban (OR 1.45, 95% CI 1.27;1.67), and intermediate urban-rural neighborhoods (OR 1.46, 95% CI 1.30;1.65) was associated with higher odds for GAD, when compared to living in rural neighborhoods (Table 5). When adjusted for neighborhood income, the association for semi-rural and GAD also became statistically significant (OR 1.13, 95% CI 1.01;1.26), while the associations of the other categories remained similar. Adjustment for demographic and SES variables, as well as smoking and physical activity, only changed the results marginally.

**Table 5:** Associations of urbanity and generalized anxiety disorder estimated from logistic multilevel models (n = 71,536).

	Model 1		Model 2		Model 3		Model 4		Model 5	
	OR	95% CI	OR	95% CI	OR	95% CI	OR	95% CI	OR	95% CI
Urbanity										
Rural	reference		reference		reference		reference		reference	
Semi-rural	1.09	0.97;1.21	1.13	1.01;1.26	1.12	1.01;1.25	1.15	1.03;1.28	1.14	1.03;1.27
Intermediate urban-rural	1.46	1.30;1.65	1.46	1.31;1.64	1.44	1.29;1.61	1.46	1.31;1.63	1.44	1.29;1.61
Semi urban	1.45	1.27;1.67	1.43	1.25;1.64	1.38	1.21;1.58	1.39	1.22;1.59	1.36	1.19;1.55
Urban	1.58	1.35;1.84	1.53	1.31;1.77	1.37	1.18;1.60	1.52	1.30;1.78	1.47	1.26;1.72

Abbreviations: OR: odds ratio; 95% CI: 95% confidence interval. model 1: unadjusted. model 2: adjusted for neighborhood income. model 3: adjusted for neighborhood income, sex, age. model 4: adjusted for neighborhood income, sex, age, education, household equivalent income. model 5: adjusted for neighborhood income, sex, age, education, household equivalent income, smoking, physical activity.

## Discussion

Our results suggest a differential health impact of urbanity according to type of disease. Living in an urban environment appears to be beneficial for cardiometabolic health, but may be detrimental to respiratory function and mental health. These associations were mostly independent of neighborhood and individual socioeconomic status, and other covariates. This suggests an effect of the residential environment itself, as opposed to health differences originating from demographic differences.

A strength of this study is the large study population and the objectively measured health outcomes. The fact that we used more than one health outcome enabled us to investigate the relationship between degree of urbanity and health in a much broader sense. Furthermore, by using multilevel models, we accounted for the clustering of participants in neighborhoods. We also accounted for individual and neighborhood level indicators of socioeconomic status. This enabled us to investigate urban-rural health differences independent from individual and neighborhood socioeconomic status.

A limitation of this study is the missing data for lung function. Missing data were imputed, and analyses were undertaken with the original dataset and the imputed dataset. Since conclusions based on the original dataset and the imputed dataset did not differ, we assume that the missing data did not influence our results. Regardless of the missing data, still a large study sample could be used. Furthermore, although we aimed to investigate the relation of urbanity and health in a broader view, we acknowledge that next to lung function, metabolic syndrome, MDD and GAD, also other diseases are important. We chose to study these particular disease because each of them can lead to a large burden of disease and because they could be measured either objectively or with a standardized and validated interview.

Mechanisms that may explain urban-rural health differences are the breeder and the drift hypotheses. The breeder hypothesis states that health differences may arise because of the environmental factors to which people are directly exposed [24]. Examples are air pollution [25], traffic noise [26] and noise annoyance [27], stress [28], overcrowding, and social isolation [8]. Spatial variation in behavior may also explain these differences. Urban living can evoke certain (un)healthy lifestyles, such as smoking, physical activity, and alcohol use, and may influence diet choices [29,30]. With the drift hypothesis, it is thought that selection processes result in urban-rural health differences. People with certain health related characteristics may move to or from specific places [24], for example because they have a chronic disease, and want to live close to healthcare facilities. These mechanisms could help explain the associations found in the current study. We found a negative relation between urbanity and lung function, which may be due to, for example, higher exposure to pollution associated with city living. Our findings suggest a potential role for smoking or physical activity, since the association between lung function and urbanity attenuated after adjustment for these lifestyle factors. Similar findings were reported in other studies [7,31]. In contrast, living in urban neighborhoods was associated with a lower prevalence of metabolic syndrome, when compared to living in rural neighborhoods. We also evaluated the individual components of metabolic syndrome and their distribution among degree of urbanity. In line with our expectations, mean SBP, DBP, fasting blood glucose, triglycerides, waist circumference and BMI increased as urbanity decreased, and HDL cholesterol decreased as urbanity decreased. One explanation may be that people in rural areas are less physically active compared to their urban counterparts [32]. Many facilities in rural areas are often not within walking or cycling distance, resulting in car use instead. Also the absence of broad walking and cycling lanes and street lights makes rural areas less attractive for being physically active [33,34]. However, associations between urbanity and metabolic syndrome found in our study could not be explained by the number of days that people are physically active. Finally, residents of urban areas had an increased risk for developing MDD and GAD, which was in accordance with the conclusions of a meta-analysis [8]. Again, (in)direct exposure to environmental factors [35], or selection processes may potentially explain urban rural variations in psychopathology [8].

This study suggests a differential health impact of urbanity according to type of disease. Future research should investigate which underlying mechanisms explain the differential health impact of urbanity. Spatial planning may facilitate behavior change in order to promote health, for example through the provision of cycling infrastructure. In addition, when it becomes more clear which aspects of the environment are threatening to our health, policy makers can diminish harmful effects by introducing legislation. The introduction of smoke-free legislation has for example resulted in substantial benefits for public health [36]. Furthermore, identification of susceptible populations is relevant for policy makers in order to initiate prevention strategies that are tailored to the population and their environment [37].
